# Home automation using general purpose household electric appliances with Raspberry Pi and commercial smartphone

**DOI:** 10.1371/journal.pone.0238480

**Published:** 2020-09-22

**Authors:** Imran Ashraf, Muhammad Umer, Rizwan Majeed, Arif Mehmood, Waqar Aslam, Muhammad Naveed Yasir, Gyu Sang Choi

**Affiliations:** 1 Department of Information & Communication Engineering, Yeungnam University, Gyeongbuk, Gyeongsan-si, Republic of Korea; 2 Department of Computer Engineering, Khwaja Fareed University of Engineering and Information Technology, Rahim Yar Khan, Pakistan; 3 Department of Computer Science & Information Technology, The Islamia University of Bahawalpur, Bahawalpur, Pakistan; National Textile University, PAKISTAN

## Abstract

This study presents the design and implementation of a home automation system that focuses on the use of ordinary electrical appliances for remote control using Raspberry Pi and relay circuits and does not use expensive IP-based devices. Common Lights, Heating, Ventilation, and Air Conditioning (HVAC), fans, and other electronic devices are among the appliances that can be used in this system. A smartphone app is designed that helps the user to design the smart home to his actual home via easy and interactive drag & drop option. The system provides control over the appliances via both the local network and remote access. Data logging over the Microsoft Azure cloud database ensures system recovery in case of gateway failure and data record for lateral use. Periodical notifications also help the user to optimize the usage of home appliances. Moreover, the user can set his preferences and the appliances are auto turned off and on to meet user-specific requirements. Raspberry Pi acting as the server maintains the database of each appliance. HTTP web interface and apache server are used for communication between the android app and raspberry pi. With a 5v relay circuit and micro-processor Raspberry Pi, the proposed system is low-cost, energy-efficient, easy to operate, and affordable for low-income houses.

## Introduction

The Internet of Things (IoT) refers to the objects that are distinctively identifiable in the virtual representation of cyberspace. It serves as a distributed network comprising of many components that are uniquely identifiable and can communicate and interact with other entities. IoT aims at processing the information and developing methods comprising RFID [1], sensing machines, smart technology, and similar other technological advancements. Predominantly, IoT has not used alone, it is aided by complementary technical developments that enhance its capabilities to bridge the gap between the virtual and physical world [2, 3]. Over the last few years, IoT sensors have been deployed in a rich variety of applications like natural disaster prediction, home surveillance, and smart farming and home automation, etc. [4–6]. Home automation (smart homes) is one of the potential application areas of IoT that has got rapid interest and growth recently. Smart devices that can sense events and translate them into meaningful data can serve to substantially maximize safety, and security and greatly improve the comfort and quality of human beings. Today, humans indulge in a busy life and desire for ease and facility in every aspect of life. The first thing that strikes our mind if we talk about the ease of people is home automation.

**Fig 1:**
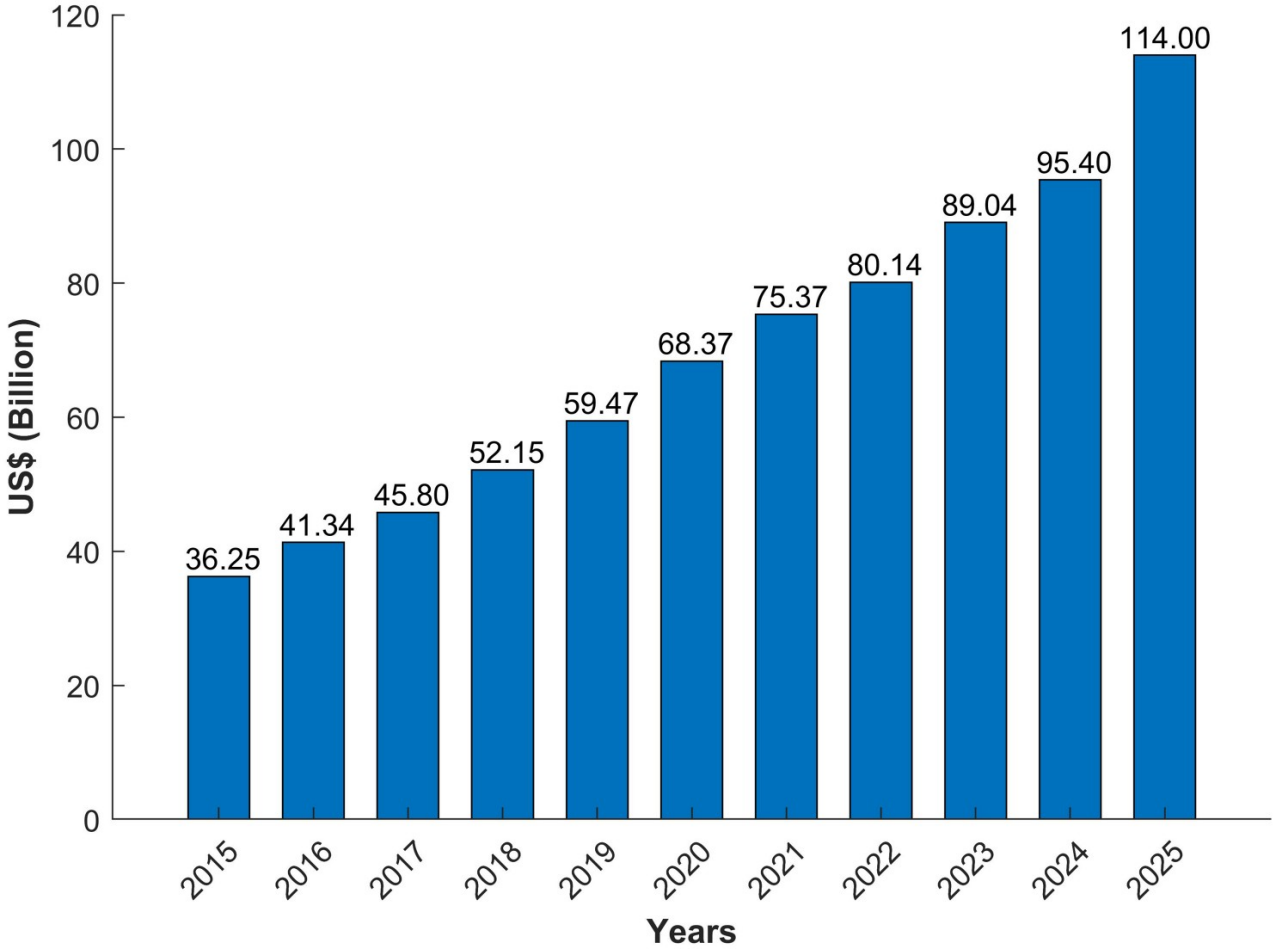
Global home automation market size for 2015 to 2025.

Home Automation term is used to describe the working of home appliances such that the human interaction is as minimized as possible [7–10]. By removing human involvement in such mechanisms, they are replaced with programmed systems and applications to control the appliances [11]. Home automation can be simply having control over few elements or it can be largely automated controlling any appliance plugged into electrical power. The popularity of home automation has increased rapidly during recent years. It is expected to grow further in the coming years and capture a huge consumer market as shown in Fig 1. A report from [12] shows that the home automation industry will have a share of 114 billion US dollars by 2025. Home automation focuses not only on the automated remote control of home appliances but optimizing the use of such appliances to save energy as well. People like the comfort of maintaining and changing the status of appliances from anywhere in the world using remote access. Security, lighting, HVAC (Heating, Ventilation, and Air Conditioning) & energy management, and smart kitchen are among the most desirable automation elements in home automation [13].

Home automation systems may be in the form of individual control devices, distributed control systems, and centrally controlled systems [14]. Individual control devices, the simplest of the home automation devices, are programmable devices that can be set to user preferences like thermostats, lighting controls, and occupancy sensors, etc. Distributed and centrally controlled systems, on the other hand, are home automation systems that can communicate and control the individual devices remotely. The difference between the distributed and central systems is that of the control whereby the former has no central controller and latter has a controller like a mobile or computer to control all the devices centrally. The downside of a centrally controlled system is the failure when the controller fails [14].

Commercial home automation systems are expensive due to custom equipment, components, and installation cost. An alternative approach is to utilize open-source platforms and IoT sensors to do customized home automation to meet the user’s own needs. There are two major problems with this approach: IP-based devices’ cost and user’s lack of expertise to operate the open source solutions for home automation. IP-based devices are expensive and home automation requires the installation of several such sensors that are out of reach of low-income houses. This paper presents an approach that can provide a low-cost home automation solution for such homes. The proposed system is built on ordinary home appliances and makes use of Relay Circuit Pack with Raspberry pi 2B to control these appliances. The term “ordinary electrical appliances” refers to those appliances that are readily available in the market. Such devices are without any special IP connector or IoT circuit. These appliances cost much less than the special IP-based smart devices like Phillips hue bulb. The system allows the user to check and change the status of electronic home appliances and the working state of sensors. Appliances include common lighting, heating, ventilation, and air-conditioning. It provides the online status of various appliance that enables the user to control and set the appliance’s usage accordingly to save the energy. The control is provided through an Android built smartphone application that is connected to the Azure cloud database. The local network provides control over the appliances when the internet is not available. An Azure cloud database is used for data logging that restores the appliances to a previous state if the system fails. The smartphone app lets the user to custom build the house on the smartphone analogous to the actual house and place each appliance that needs to be controlled. The proposed system is easy to use with the provided GUI (Graphical User Interface) and provides full control over electrical appliances. In summary, the proposed system makes the following contributions:

- A microprocessor-based (Raspberry Pi) based home automation system is presented that can sync the data to the Microsoft Azure cloud server, store the recent state of the appliances and resume the state in case of failure.
- Data logging is provided that can help the user to optimize the performance of appliances and save energy.
- Contrary to expensive IP-based devices, the proposed system transforms ordinary electrical appliances like tube light, fan, etc., to remotely controllable devices.
- A smartphone app is designed that helps the user to design the automated home by drag and drop modules.

The proposed system makes use of Microsoft Azure as the cloud data storage system. Microsoft Azure is the fastest-growing Cloud Infrastructure Platform and superior over other cloud services in terms of cost, flexibility, security, hybrid capability, and API (Application Programming Interface) management. Furthermore, the prime reason for using Microsoft Azure as the cloud service is its facility to provide big data insights using Apache Hadoop. It also provides the data in the refined form of excel for data mining. Consequently, an IoT based smart home automation system can be operated without human intervention.

The rest of the paper is organized in the following manner. Section 1 describes the research work related to the current study. The components and functionality of the proposed system are discussed in Section 1. Results are given in Section 1, while Section 1 contains the discussions and conclusion.

## Related work

Home automation has been a topic of great interest for the last few years, hence an amplitude of research works, as well as, a bunch of commercial systems can be found. We will discuss a few research works that are closely related to the current study. We divide the home automation systems into categories depending on the network used for automation like Arduino, and ZigBee, etc.

A Wi-Fi-based HAS (Home Automation System) is presented in [15] to control the electrical appliances with a smartphone app. The system is based on Wi-Fi Module ESP8266 which is connected with an Arduino Mega 2560 module. The Relay board is used to control the power of electrical appliances like fans and bulbs. Other sensors placed in the models are a fan, humidity sensor, motion sensor, buzzer, and temperature sensor. A smartphone app controls the off and on operations of these sensors in a model house that is a miniature template of a house. Similarly, authors in [16] present a system for home automation that is based on Arduino UNO, Raspberry Pi, and nRF24L0+ wireless transceiver. A fan, light, door, and a motion sensor are placed inside the house and controlled remotely. Another IoT HAS is presented in [17] that leverages Arduino UNO MCB (Micro-controller Unit Board) and ESP32 Module that operates on 2.4 GHz. Four appliances have been controlled by the system including the light bulb, gas sensor, temperature sensor, and Liquid Crystal Display(LCD). The system is a web-based solution for HAS and does not have a smartphone app to control the appliances. In the same fashion, authors in [18] propose the use of Arduino for HAS controlled with a web page. Additionally, electrical appliances and devices can be controlled with a Bluetooth based smartphone app. The IP base temperature, light, gas, and smoke sensors are utilized for automation. The Google speech recognition module is incorporated as well in the proposed system for voice control. An Arduino HAS is proposed in [19] that integrates WLAN (Wireless Local Area Network) for controlling the electrical appliances. A smartphone app is available to control the light bulb, fan, motion, and smoke sensors.

The use of ZigBee has also been investigated for home automation in [20]. The authors propose the use of ZigBee and Wi-Fi network integration to control the devices placed in a smart home. Authors analyze existing systems to investigate why do these systems attract a low number of consumers. Complex and expensive architecture, intrusive installation, lack of network interoperability, interface inflexibility, and security and safety are found to be the common reasons for HAS to attract a wide audience. Later a home automation system that can be controlled by a web page, as well as the remote control, is presented. Light bulb, radiator, smoke sensor, and a ZigBee remote control has been deployed for home automation.

A globally accessible HAS is proposed in [21] that is controlled using a web page. The devices in the home are monitored by Raspberry Pi micro-controller that operates with electromagnetic relays. The interaction with Raspberry Pi is carried out with the Webiopi framework which is a weaved cloud service. A NodeMCU (node Micro-controller) based HAS is proposed in [22] that uses Wi-Fi gateway to control various devices. The sensor state and data can be updated using the Adafruit IO cloud server. Data from the deployed sensors can be collected and accessed using IFTTT (If This Then That) on a smartphone. NodeMCU has connected relays to control home appliances. Various devices are controlled via the system like lights, and fans, etc. Besides automation, various systems for surveillance and home security have also been proposed with IoT sensors [23]. Similarly, a street light control system is discussed in [24] that makes the use of Raspberry Pi.

Above-cited works are limited by many factors that limit their wide application for home automation. First, Arduino is predominantly used for home automation. Arduino and Adafruit are micro-controllers that are used for home automation. Micro-controllers are limited by function as the I/O pins are dedicated to performing various tasks. We make the use of Raspberry Pi instead which is a microprocessor and can perform complex tasks and make decisions like sync data to Microsoft Azure cloud storage for lateral use. Second, IP based devices that are used in the majority of the proposed systems are expensive. For example Philips LED smart bulb costs 10-15 US$ [25], while a smart Wi-Fi plug costs 20 US$ [26]. We turn the ordinary electrical alliances into remotely controllable devices through the proposed system and the user does not need IP-based devices. Thirdly, users are unable to design their automated home as the third party installs the appliances and provides a smartphone app for its control. Often it is expensive and time-consuming. Our proposed system facilitates the user to build his home on the smartphone using drag and drop options which is convenient and cost-effective.

## Material and methods

This section provides the details of the proposed automation systems, its components that interact to control electrical appliances and their working principles.

### Designing and decor of the smart home

**Fig 2:**
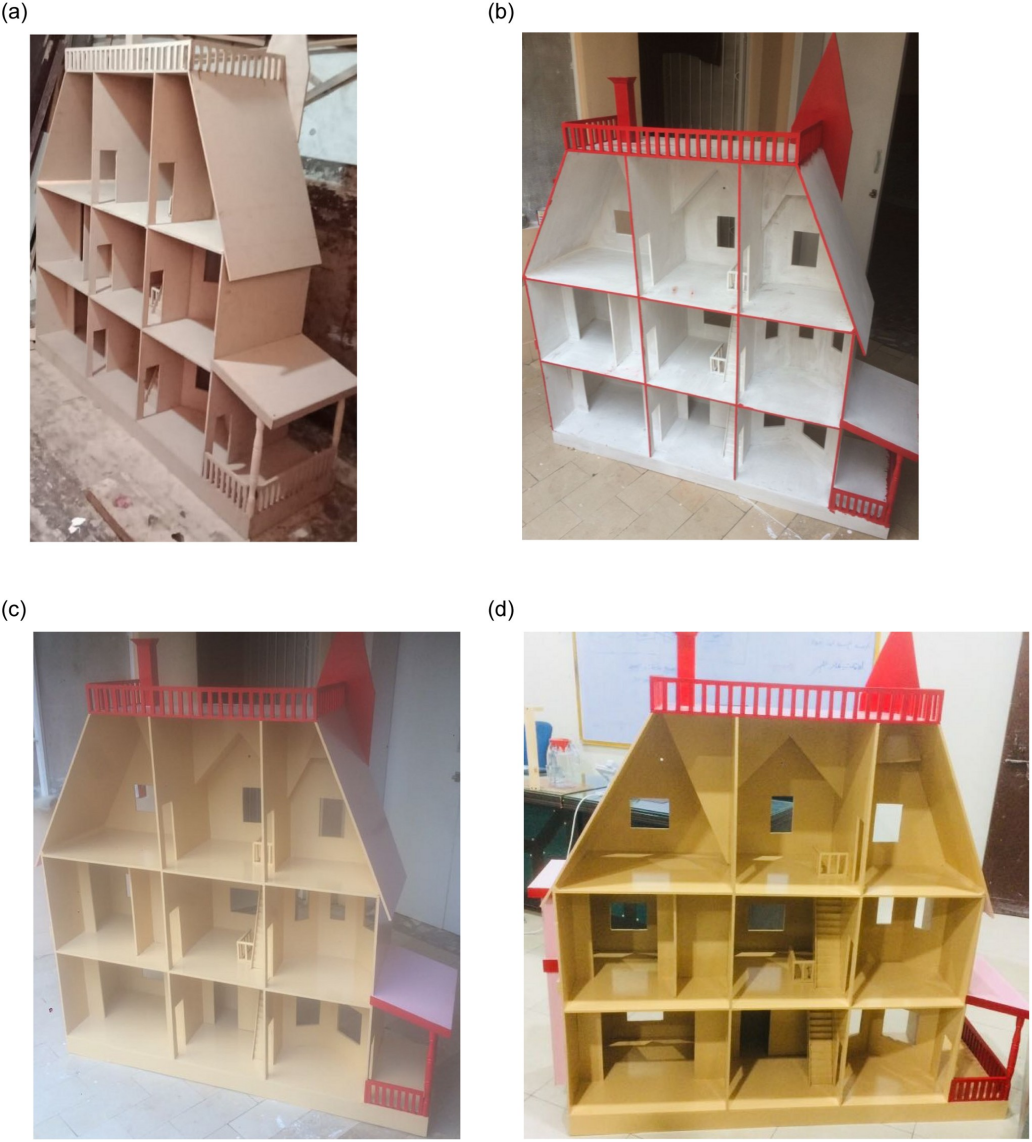
Pictures of the design process of the smart home prototype, (a) side view of initial design with the base color, (b) primer paint on the prototype, (c) first coating of the selected color, and (d) the prototype in its finished form with the selected color.

The prototype of the proposed system is designed using Autocad. The prototype comprises a ground, first and second floor each with a different set of rooms. The ground floor has one bathroom and one living room while the first floor includes a sitting room that has a place for TV and a study room. On the other hand, the second floor has a bathroom, a sitting room, and a living room. Fig 2 shows the layout and phases of smart home development. The prototype is prepared using the plywood sheets that are cut and joined to shape them for the prototype. For joining the sheets, glue and screws are used. The plywood sheets are cut according to the design made in Adobe Photoshop. A primer or preparatory color is coated on the screws and plywood boards to ensure the adhesion of the selected paint for the prototype. Three coatings of the selected color are applied on the plywood and the finished prototype is shown in Fig 2d. In its finished form, it looks beautiful and smooth. The paint ensures that the prototype is free from any damage in the case of electricity sparks and water.

**Fig 3:**
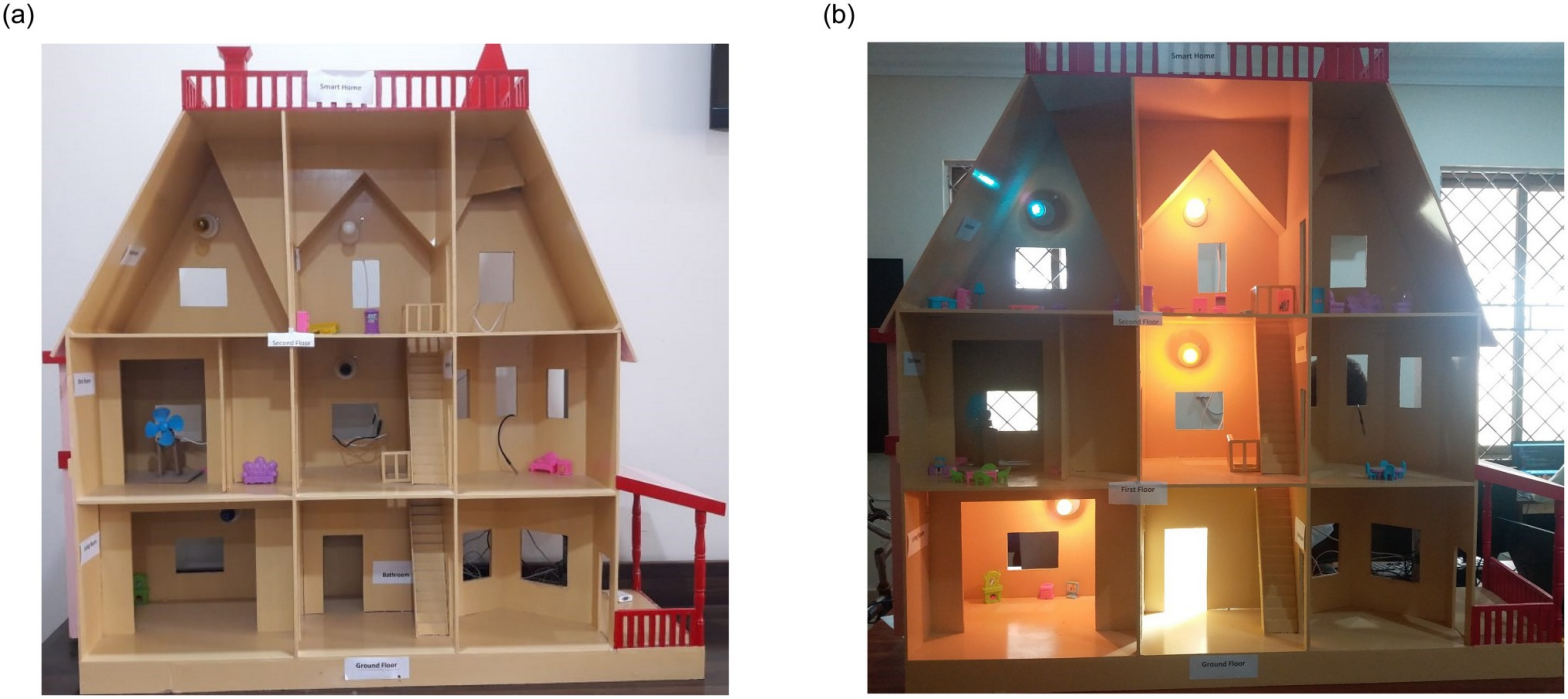
Pictures of the design process of smart home prototype, (a) side view of initial design with the base color, and (b) final prototype with installed appliances/sensors.

Once the design and decor of the prototype are complete, now it needs to be populated with the furniture and electrical appliances/sensors. Chairs, tables, and other furniture items are placed inside the living rooms, sitting rooms, study rooms, and bathrooms as shown in Fig 3a. For this purpose, various sensors and actuators are installed that are later controlled with Raspberry Pi. Fig 3b shows the picture of the final prototype with all the sensors installed in it.

### Proposed system

**Fig 4:**
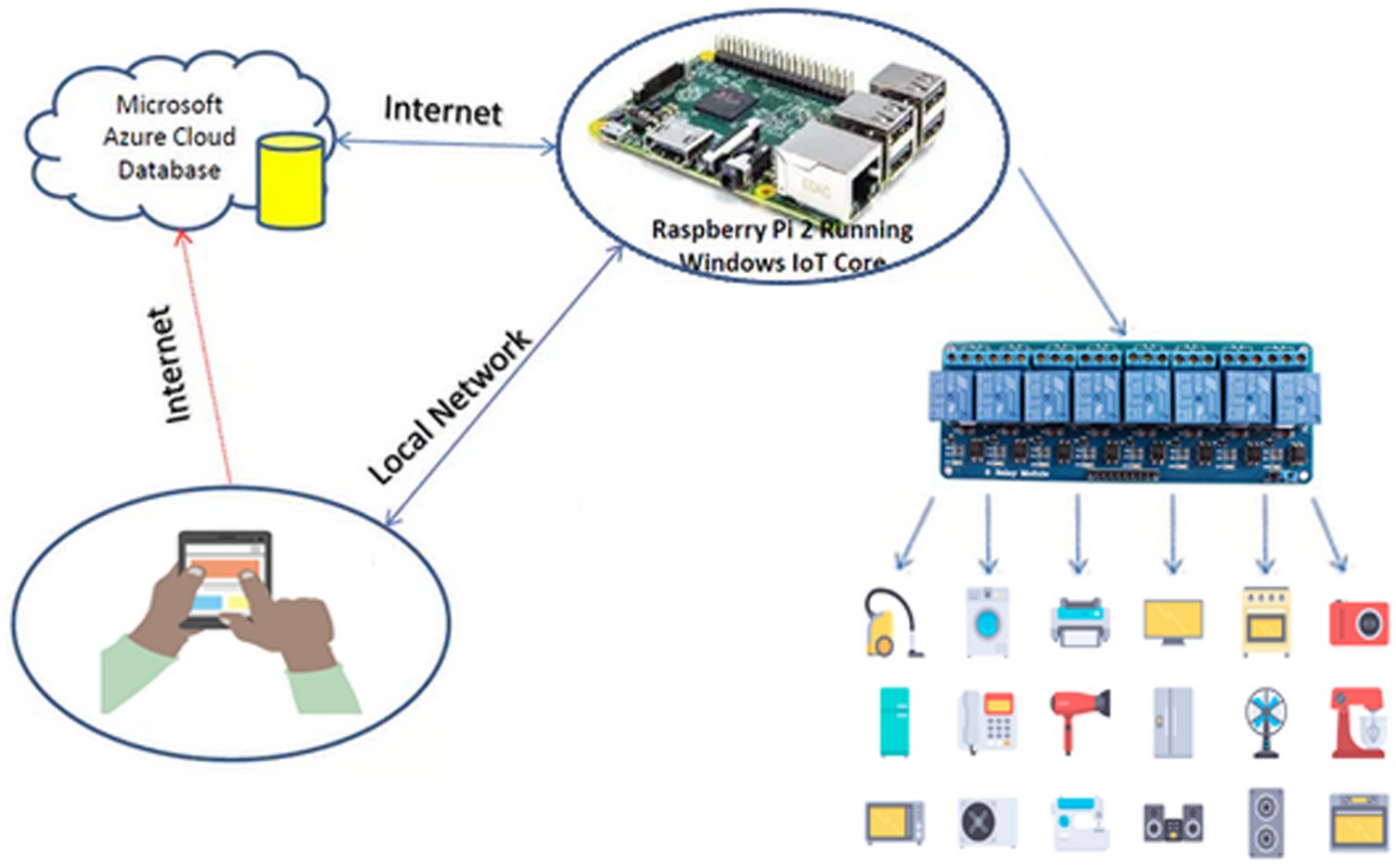
The architecture of the proposed home automation system. The proposed system comprises of four main modules. The arrows joining these modules represent the data flow while the arrow direction indicates the direction of the data flow.

Fig 4 shows the architecture of the proposed home automation system. Broadly the system is made of five components in total: a smartphone app, Microsoft Azure cloud database, Raspberry Pi, relays circuits, and electrical appliances. Arrows in the figure show the flow of data from one component to the other. Arrowhead represents the flow direction and a double-headed arrow indicates that there is two-way communication. There are two network modes in which the user can interact with the Raspberry [27–29]. If the user is inside the home or within the range of the local network, he can IoT services at a local network without connecting to the internet cloud. The term local network indicates the dynamic IP from the wireless router that is available even if the internet is not working. The proposed system can utilize this IP without any gateway involved to control the devices. This will also result in faster communication between the devices and the user smartphone app when the user is controlling locally.

The second mode of the network can be used when the user is away from home and wants to control home appliances remotely over the internet. In this case the request to the Microsoft Azure Cloud [30, 31]. On the verification of the user’s given credentials, the request is forwarded to concerning Raspberry Pi for further processing. Services for each user are handled based on credentials for which the request is being generated. The APIs are called from the cloud if the user is outside of the home network. The same APIs are also residing in the Raspberry Pi server if the user is in the home network.

Data sharing between application and server database is through a JSON (JavaScript Object Notation) file. The API’s are secured using multiple hashing techniques. The change in the state of any device is performed using Raspberry Pi GPIO (General Purpose Input/Output) [32] pins. Raspberry Pi receives the request from the server and responds to concerning devices. The database of each request generated by any individual user is maintained at the cloud server. The user can check the complete history of the processed requests on the smartphone by setting the specified duration to view the requests. Sensors installed inside the home update their states continuously after 30 s and respond to the change to the Raspberry Pi server. In response, the Raspberry Pi server syncs all the data to the cloud database and refreshes the data shown on the smartphone application as well.

### Hardware components

Many electronic components, as well as sensors, are utilized for the current system. A complete list of components along with the description is given in Table 1.

**Table 1:** Electronic component with specification details used in the project.

Components	Specifications
Raspberry pi 2B	40 GPIO pins, 1GB RAM, A 900MHz quad-core ARM Cortex-A7 CPU, Operational voltage 7-12v
Relay Circuit Pack	The 5v operational 8-relays circuit pack
L293D motor control shield [33]	Supply-Voltage Range: 4.5–36 V, Output current: 600 mA/channel
Smartphone Mobile	Android Supported
DS18B20 Temperature Sensor [34]	temperature range: −55°C to 125°C, (−67°F to + 257°F)
LM393 LDR Sensor [35]	Digital switching outputs (0 and 1), External 3.3V-5V Vcc
MQ2 Smoke Sensor [36]	Combustible Gas, Smoke

#### Raspberry Pi 2B

The Raspberry Pi comprises of several small single-board computers often used at schools to teach basic computer science concepts in developing countries. It contains A 900 MHz quad-core ARM Cortex-A7 CPU (Central Processing Unit) processor along with 1 GB of RAM (Random Access Memory). Arm Cortex-A7 is built on the energy-efficient 8-stage pipeline and has an integrated L2 cache designated for low-power and reduced latency [37]. It supports 100 MBPS Ethernet and contains 4 USB ports along with 40 GPIO pins. Full HDMI (High Definition Multimedia Interface) support with camera and card interface is available as well as Micro SD card. It contains 3.5 mm audio jack and composite video support too.

Raspberry Pi is selected over Arduino for several reasons. First, Arduino is a microcontroller that can run one program at a time. On the other hand, Raspberry Pi is a micro-processor or general-purpose computer that can run multiple programs. Second, although Arduino is simple and easy to use than that of Raspberry Pi, it is often used for simple tasks like measuring temperature, opening a door, etc. Raspberry Pi is good when we have to perform multiple tasks involving calculations and decision making. For our system, Raspberry Pi is more suited to the control over appliances from various users. Moreover, the availability of an SD card slot and a large number of GPIO pins makes it suitable for systems where a variety of appliances are to be controlled.

#### 5v relay circuit

**Fig 5:**
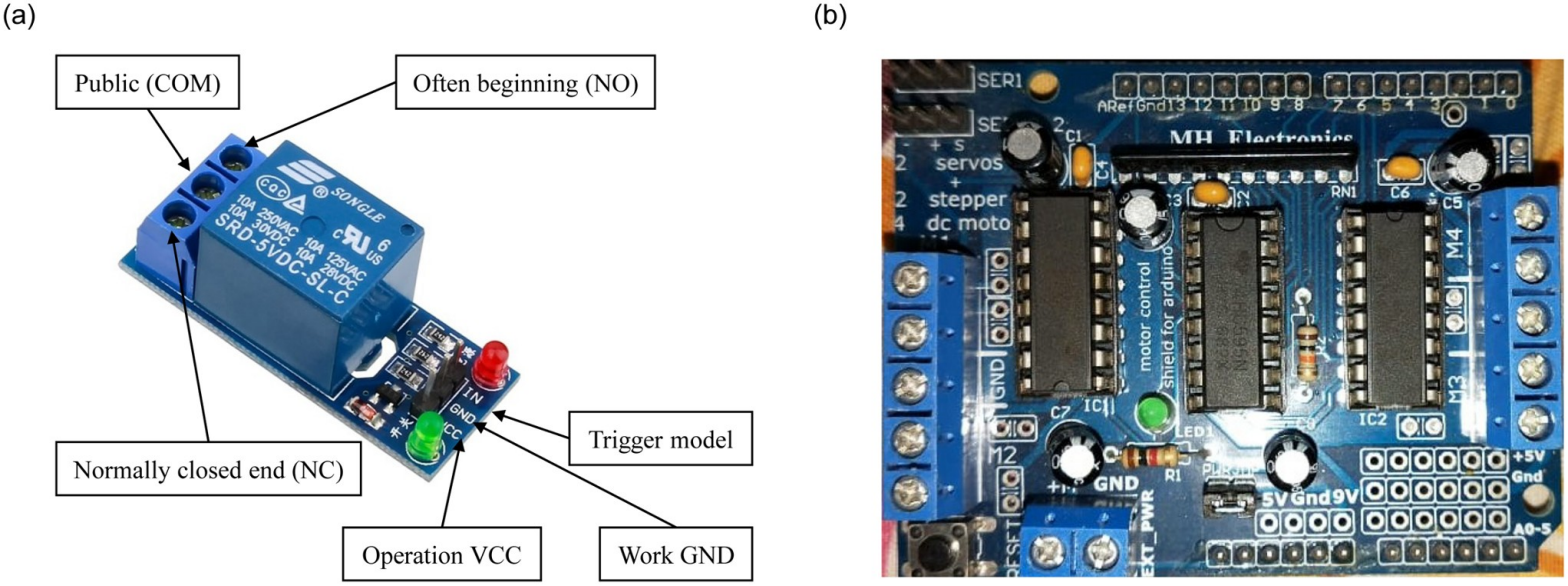
Pictures of the sensors used in the current study, (a) picture of the relay used in the system along with the details of its important parts, (b) picture of the motor used in the system.

A relay is an electrically operated device. It has a control system (also called input circuit or input contactor) and a controlled system (also called output circuit or output contactor). It is frequently used in the automatic control circuit. To put it simply, it is an automatic switch to controlling a high-current circuit with a low-current signal. Fig 5a shows the picture of the relay that is used for the current system.

#### L293D motor shield

L293D is a monolithic integrated, high voltage, high current, 4-channel driver [23]. It can be used for DC motors and power supplies of up to 16 volts. It shows that it has enough capability to support pretty big motors. It offers a maximum current of 600 mA per channel. L293D chip is alternatively known as an H-Bridge. The H-Bridge is typically used in an electrical circuit to apply voltages across a load in either direction to an output, e.g. motor. Fig 5b shows the motor shield used in our system.

#### DS18B20 temperature sensor

**Fig 6:**
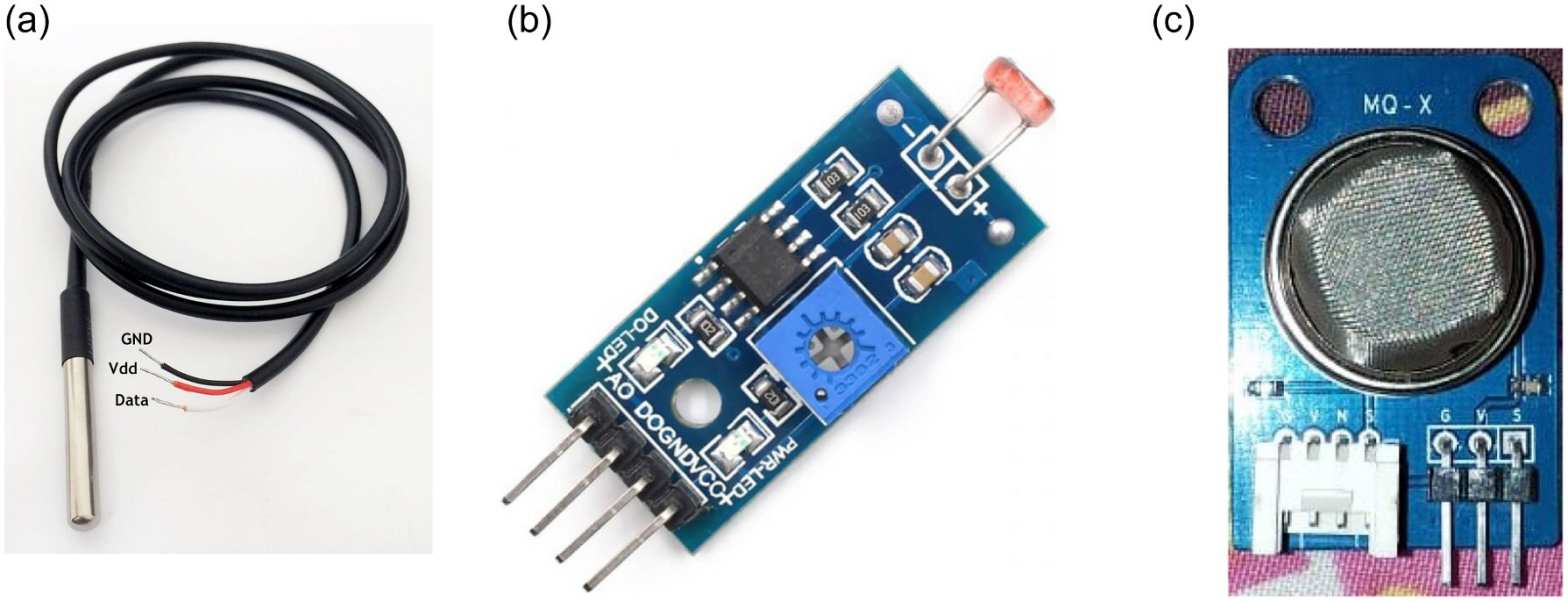
Pictures of the sensors used in the current study, (a) temperature sensor, (b) light sensor, and(a) smoke sensor.

The DS18B20 temperature sensor is a 1-wire digital temperature sensor [38] that can measure the temperature of an environment. It communicates on a common bus that can connect several devices and read their values using just one GPIO pin of the Raspberry Pi. Fig 6a shows the temperature sensor used in our system.

#### LM393 LDR sensor

The LM393 is a photo-resistor light sensor that has both analog and digital outputs [39]. The digital output uses a trim potentiometer that can set a trigger light level. In the current system, we use the analog output to measure the light intensity level. Fig 6b shows the light sensors used in the current system.

#### MQ2 smoke sensor

MQ2 is one of the widely used gas sensors in the MQ2 sensor series [40]. It is a Metal Oxide Semiconductor (MOS) type gas sensor also known as Chemi-resistors because the detection is based on the change of resistance of the sensing material when the gas comes in contact with the material. Using a simple voltage divider network, concentrations of the gas can be detected. MQ2 gas sensor works on a 5V DC and draws around 800 mW. It can detect LPG (Liquefied Petroleum Gas), smoke, alcohol, propane, hydrogen, methane, and carbon monoxide concentrations between 200 to 10,000 ppm (parts-per-million). Fig 6c shows the gas sensor used for the current study.

### Connecting the appliances/sensors with relay and Raspberry Pi

**Fig 7:**
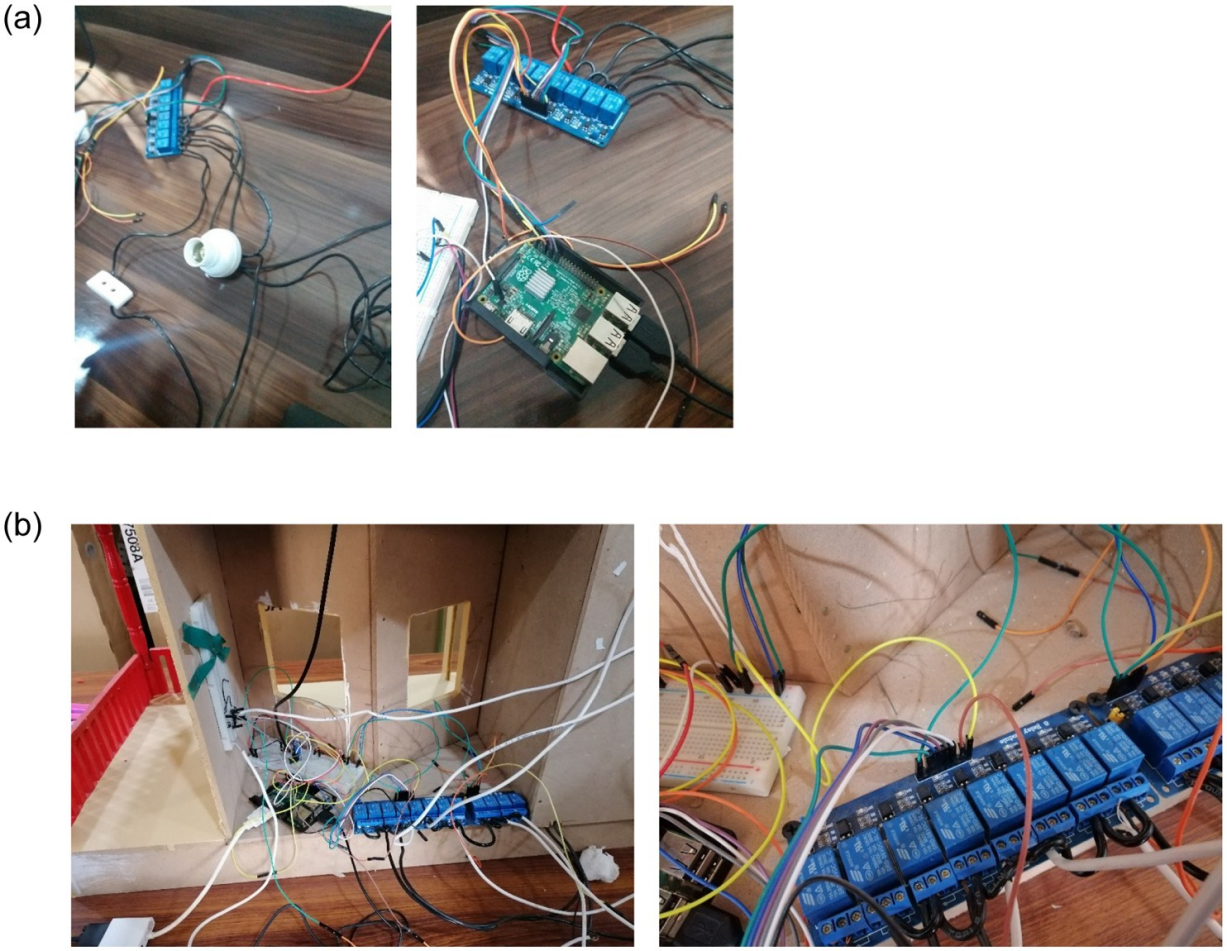
Pictures of wire installation of sensors, relay and Raspberry Pi, (a) wire installation for initial testing, (b) final wiring and placement in the smart home.

Initially, the proposed system is tested with a couple of devices like light bulbs to evaluate its performance. Wires are attached that link the appliances and relay units with the Raspberry Pi as shown in Fig 7a. Once the system is tested for its initial performance, it is put to the final use by placing it inside the smart home. The system is initially controlled using a laptop and later a smartphone app is designed to control it remotely. The wire installation and placing the controllers in the smart home is shown in Fig 7b.

### Software components

For the development of the mobile application, there are many platforms available like Symbian, Android, iOS, and Windows Mobile. We consider the Android operating system for the current system as approximately 74.16% of the total smartphones operate on Android [41]. The android operating system is supported on the majority of devices and we can provide the home automation service to a large community if we develop the system on Android. Java language with Android SDK (Software Development Kit) has been used for the development and implementation of the home automation smartphone application.

#### Android studio

Android Studio 3.4.2 build # AI-183.6156.11.34.5692245 is used for the development of the smartphone app. Android Studio supports all development tools such as a debugger, libraries containing built-in functions for various tasks, and handset emulators [42]. Volley library is used for sensor services while the ‘material design’ library is used to make the application more interactive.

#### Server side scripting

Server-side scripting is exercising scripts on the server-side to provide a customized interface for clients. Such scripts generate a response for individual client’s requests. Scripts are deployed on two servers for the current system: Azure cloud and Raspberry Pi. For this purpose, Linux, Apache, MySql, and PHP (LAMP) is used to provide the backend functionality.

## Working methodology

**Fig 8:**
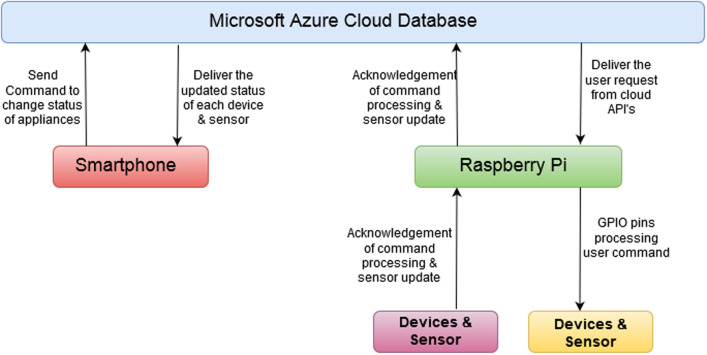
The process followed when the user controls the devices remotely during Azure cloud database server.

Microsoft Azure cloud database server and Raspberry Pi are the two most important components of the proposed system that controls the communication of the user requests and sensors response. These components work concerning two scenarios of the user’s location. In the first scenario, the user is not in the vicinity of the home. The user wants to control the devices remotely over the internet and the local network is not used for this purpose. This scenario involves using the Microsoft Azure cloud database. Each request from the user is sent to the cloud using the smartphone app. The user’s credentials are authenticated and then the request(s) is forwarded to Raspberry Pi. The specific APIs are called to fulfill the user’s request. On the reception of requests from Azure cloud, Raspberry Pi executes the commands by turning on/off the dedicated GPIO pins for the sensors that users requested. If executed successfully, acknowledgment is sent to Azure with the current status of the devices. Fig 8 shows the process that is carried out for a remote request. Algorithm 1 monitors and automate the appliances/sensors placed in the proposed smart home.

**Fig 9:**
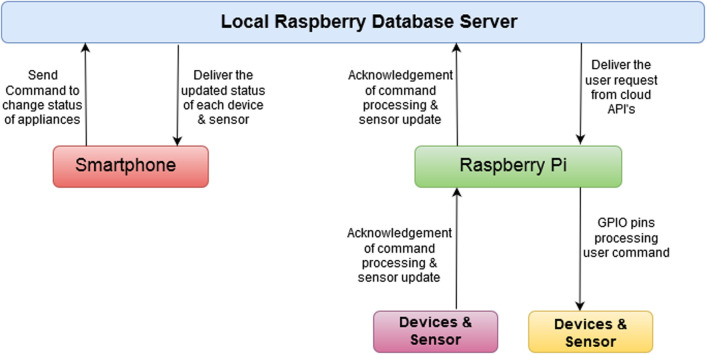
Raspberry Pi executes user’s requests when the user is inside home and Azure database server is not involved.

Raspberry Pi server is responsible for carrying out the instructions so that the user requested devices can be operated as desired. In the second scenario, when the user is inside the home and connected to the same network to which raspberry pi is connected, all the requests go directly to the Raspberry Pi server. It leads to fast processing and execution of requests as the cloud is not involved. Fig 9 shows the process that is followed when the user is inside the automated home.

The implementation of home automation is done in such a way that the status of the devices is recorded periodically for the recovery. The backup is done for both the discussed scenarios on the cloud server. So in case of Raspberry Pi restart or electricity failure the most recent state of the devices is saved and devices can be set again to the state before the restart. This involves the use of a database server that maintains the state of each device. The last state record of each device is fetched and set again to each device accordingly.

**Algorithm 1** Monitoring and controlling of smart home.

**Require**: Monitoring home conditions and controlling appliances locally and remotely

**Ensure**: Real-time monitoring (temperature, gas, light sensor), and remotely control home appliances (HVAC, any ON/OFF devices)

 **Define**: Wi-Fi Access Point Username/PW and static IP

 **Define**: Raspberry Pi local & Microsoft Azure Cloud Server

 **Define**: GPIO, GND, PWM, SPI I2C pins for Relay board // Used to switch appliances/devices (lights, motor, fan, and buzzer, etc.)

 **Define**: API’s. Volley Library for sensors // Get devices/sensors data

 *D* ← *Darkness* *value* // From LDR sensor (Threshold value is 120lux)

 *T* ← *Temperature* *value* // From (DS18B20 threshold value 35 centigrade)

 *G* ← *Gas* *value* // From MQx gas sensor

 Set threshold SENSORS values: DTH, TTH, GTH

 Initialize IoT@HoMe // All appliances getting its last state(ON/OFF) from the server database

 Sensors are connected to the Raspberry PI

 **Raspberry PI** server is connected via Wifi Access Point

 APis and Volley Library acquire the sensors data to local server

 **for** each round **do**

  Get L, T, G and D

  Upload data to **Raspberry Pi and Azure Cloud Server** over **Wi-Fi**

  Update **status** of sensors/appliances on **Raspberry Pi Server**

  Synchronize data to **Mobile Application** from Server

 **case** (LDR):

  **if** (*D* ≥ *DTH*) **then**

   Switch ON lights

  **else**

   Switch OFF lights

   break;

  **end if**

 **case** (TEMP):

  **if** (*T* ≥ *TTH*) **then**

   Switch ON fan/AC

   Notify user via API’s “Temp. is High! Ventilation Mode is ON”.

  **else**

   Switch OFF fan/AC

   break;

  **end if**

 **case** (MQx):

  **if** (*G* ≥ *GTH*) **then**

   Switch ON Ventilation FAN & Switch ON Buzzer

   Notify user via API’s “Gas leakage! Ventilation Mode is ON”.

  **else**

   Switch OFF V_ fan

   break;

  **end if**

 **end for**

 User views data from devices/sensors in real-time remotely via **Mobile Application**

 Remotely control appliances via **Mobile Application**

 User views long data from all appliances in real-time remotely via **Mobile Application Logs Tab**

## Results and discussions

A smartphone app is built to operate all the electrical appliances operating in our home automation system. The smartphone app comprises of two modes of operation: admin mode and user mode.

### Admin mode

**Fig 10:**
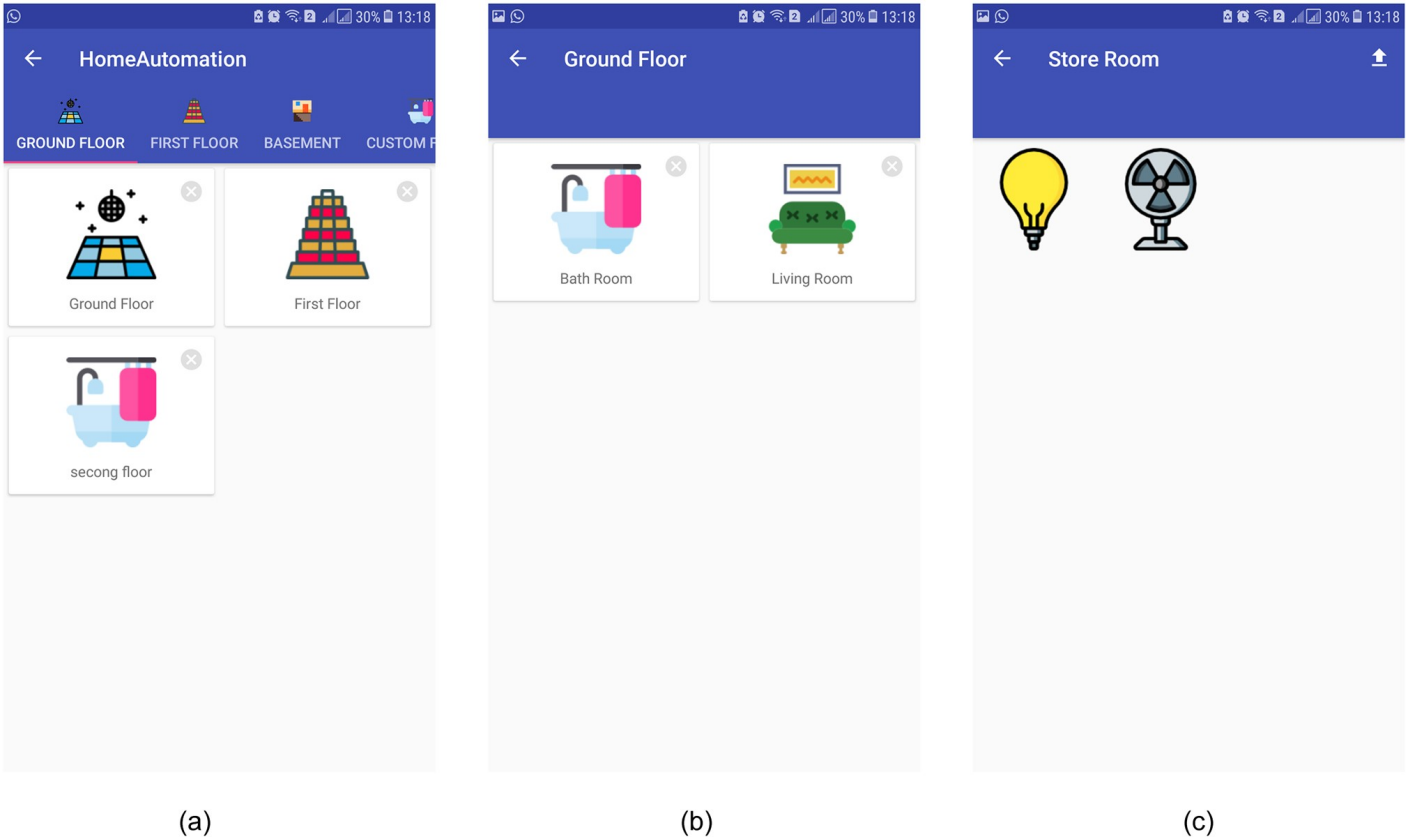
The working flow of admin mode of smartphone app. (a) Various floors are added, (b) Living and bath room are added to ground floor, (c) Appliances are added to a floor.

The Admin mode of the smartphone app provides an interactive way to build the components of home automation. It allows admin to add various kinds of the floor for home automation like the ground floor, basement, and custom floor, etc. as shown in Fig 10a. Once floors are added, rooms can be added to a particular floor like a bathroom, and a living room, etc. Fig 10b shows the screenshot of the smartphone app where the ground floor is populated with rooms. After that, electrical appliances can be put in place in a specific room, as shown in Fig 10c where a bulb and a fan are added. All such operations are through an easy drag & drop option and require a very short time. Each appliance/device placed in the home automation control app is assigned a raspberry pi PIN that controls the actual working of the electronic device in the backend.

### User mode

**Fig 11:**
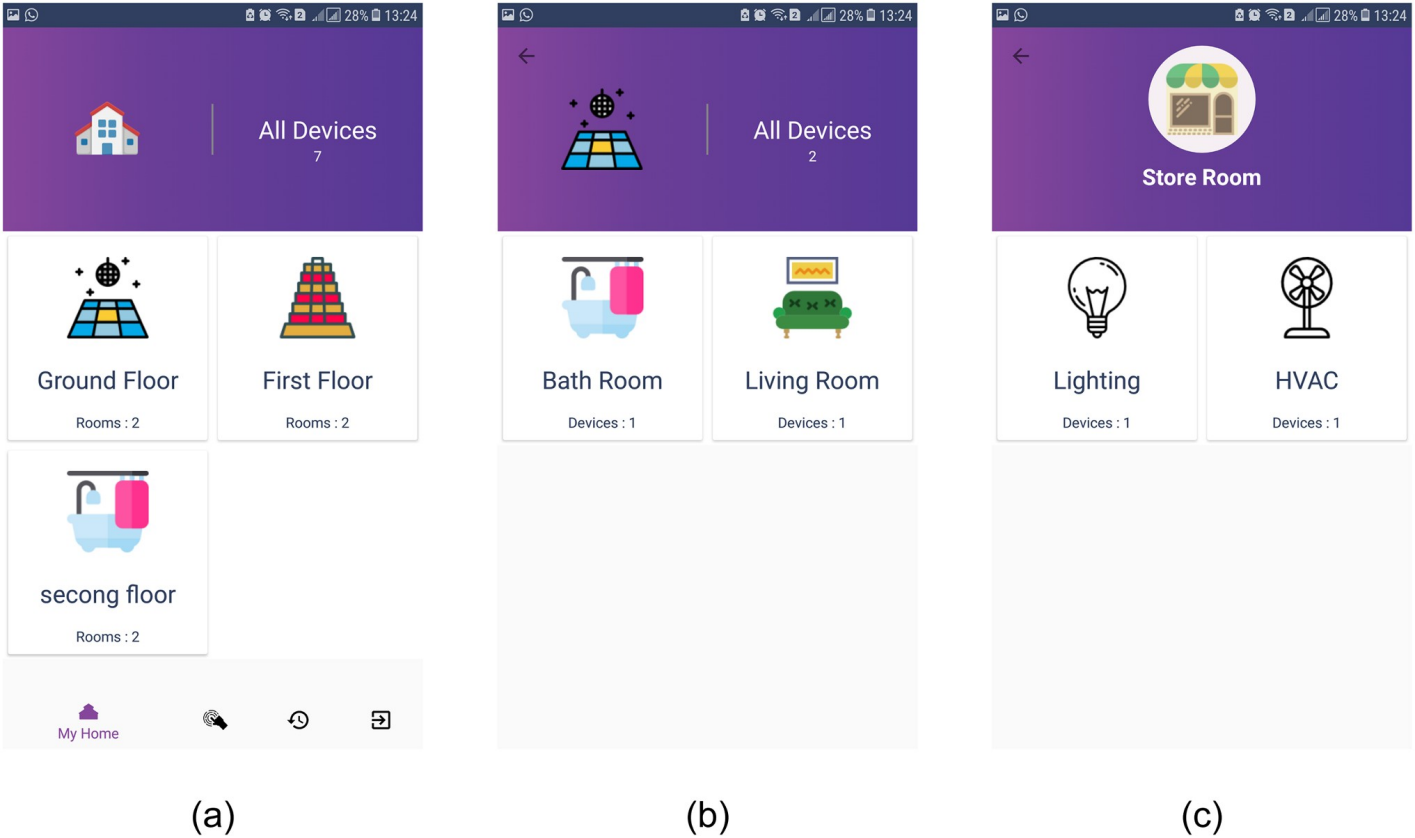
The working flow of user mode of smartphone app. (a) Various floors are added, (b) Living and bath room are added to ground floor, (c) Appliances are added to a floor.

User mode contains various functions controlled by the user of an automated home. The user is not only able to see the admin built the whole prototype but also can control the working of placed electrical appliances. For example, Fig 11a shows the floors placed in an automated home, as well as, the total number of appliances/devices (i.e., seven in this case) that can be controlled by the user. Once the user selects a particular floor, it shows the appliances put on that floor only as shown in Fig 11b which indicates one appliance is placed in the bathroom and living room each. Users can control the device after selecting the specific room. Fig 11c shows that the ‘storeroom’ has a lighting bulb and HVAC that the user can control. Additionally, data logging is provided for all the used appliances in the automated home. The purpose of data logging is to help device usage optimization to save energy. The data can be logged locally (phone) or on a server. Although smartphone possess enough data storage capacity to log the data from the home automation system. Yet, they do not offer services for efficient numerical and visual display of the data for analysis. Logging the data on the server is fruitful for data analysis. User can analyze the stored data for device usage time and optimize the performance of appliances and save energy.

### The control of devices through the app

**Fig 12:**
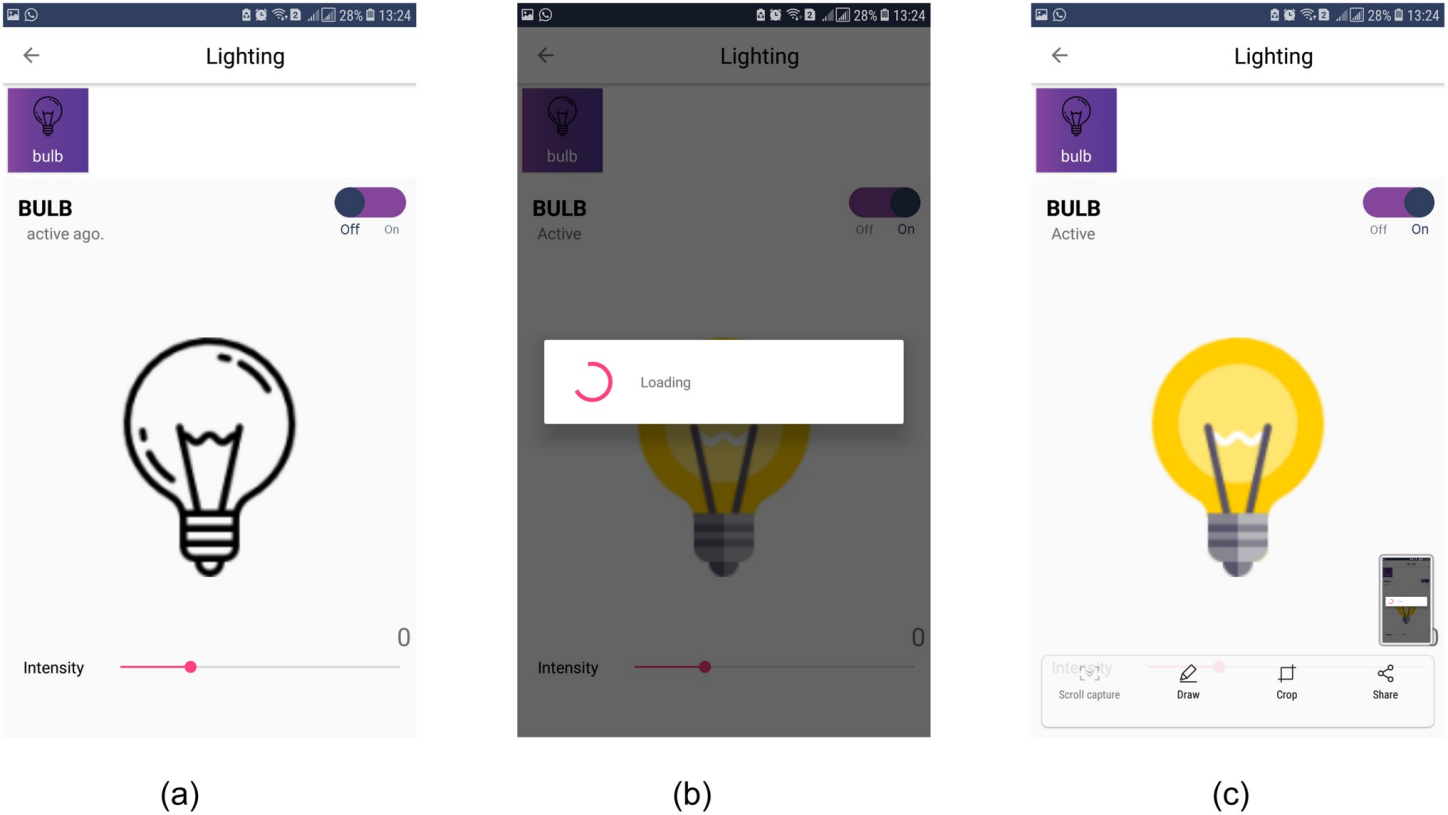
The working of changing state of light bulb. (a) Light bulb is selected to change its status, (b) Light bulb processing after turning it on, (c) Light bulb after its status is changed to on mode.

The smartphone app facilitates the users to control the appliances placed in the automated home. Nice and interactive Graphical User Interface (GUI) based control with attractive icons let the users know the status of electrical appliances. Icons are changed to show the user the current state of an electronic device with a touch active button to change state. The status of devices is shown with an active working state. For example, Fig 12 shows the working example of checking and changing the state of a light bulb from the user. Fig 12a shows that the light bulb is off. Turning the light on is shown in Fig 12b. At the bottom of light control, a brightness bar is shown as well which gives the user the option to set light intensity. Once the light is turned on with the set intensity, its color is changed as shown in Fig 12c.

**Fig 13:**
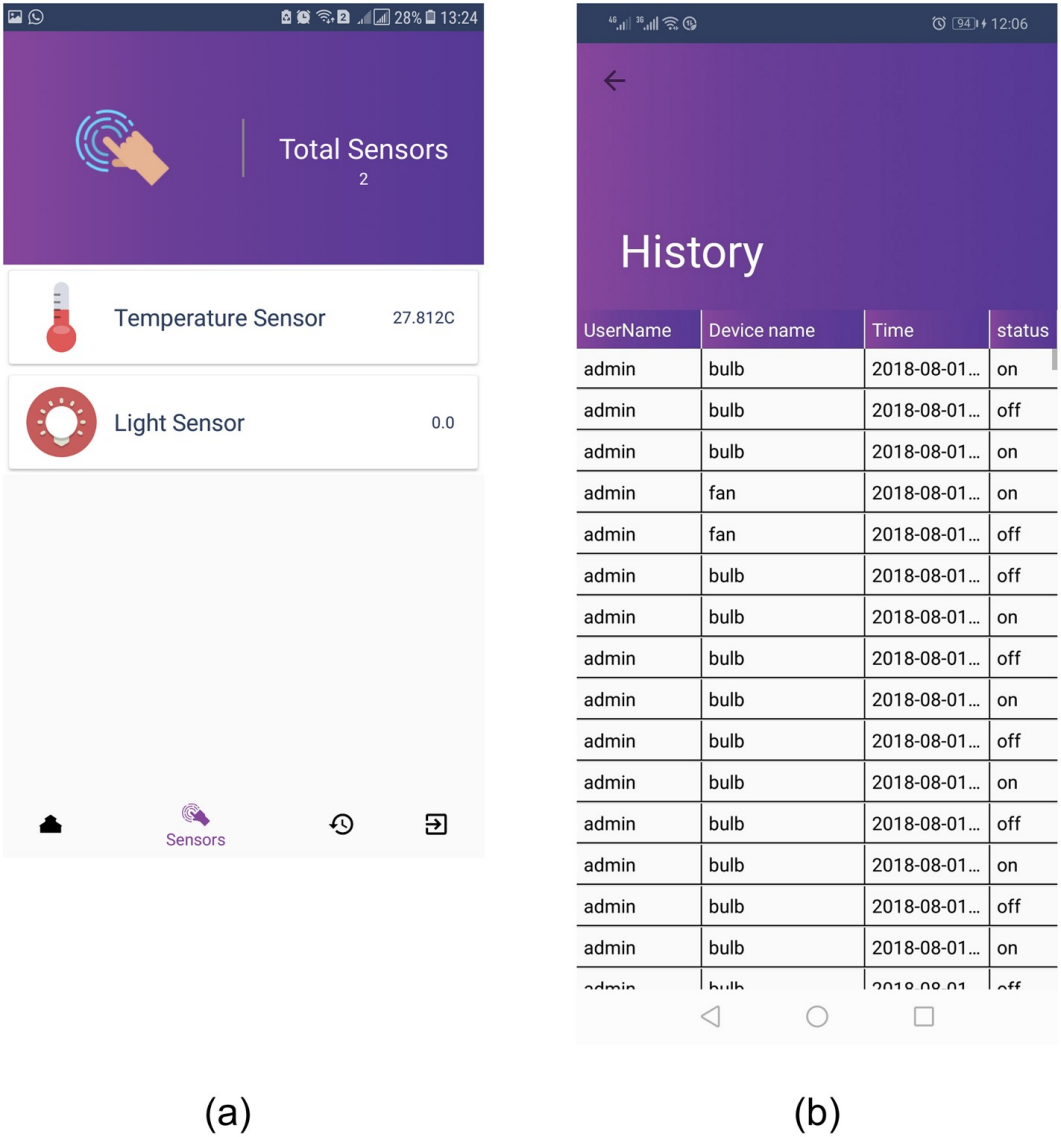
The state and history of appliances/devices, (a) state of sensors in a specific room, (b) complete history of the status of a light sensor.

User’s set appliances can be viewed to check their status, as well as, the reading each appliance. For example, Fig 13a shows that there are a total of two sensors, i.e., a temperature sensor and a light sensor placed in a specific room. Light sensor value 0.0 shows that currently daylight is active and the sensor is in the off state. The user can view the history of a particular sensor over time to optimize the performance. Fig 13b shows the complete record of the light bulb for its on and off states. The values of sensors get updated using backend triggers which refresh itself after every 30 s.

### Automatic execution of user’s set preferences

Optimization is achieved through the introduction of another function called, ‘notification’. A user receives a notification on his smartphone if any device is running for more than two hours. It works like an alarm and reminds the user of any appliances left turned on accidentally. Based on the working time of a particular appliance, a bill calculation system is in place as well that the user can use to estimate his bill based on electricity consumption. User’s set preferences are executed automatically in the proposed automation system. For example, if the user has set a temperature threshold for his comfort it will be maintained by the system. If the temperature of the home rises higher than the threshold set by the user, ventilation fans will start working automatically to lower it down and vice versa. Similarly, the light sensors are turned off and on for the measured light intensity during the day and night.

### Smartphone app for home automation system

In the light of the above-described functionality, the following are the striking differences between the built app for the proposed automation system and apps provided in other similar home automation systems.

1. Current app provides complete control to the users as well to place and control the appliances in the automated home while other apps give only partial control.
2. Sensors/appliances can be placed as per the original design of an actual home which is not possible in other apps.
3. Placing the appliances requires users to only drag and drop, while other apps involve complex handling often done by the Admin instead.
4. Besides the ON and OFF functionality of the appliances, intensity can be controlled as well, like light intensity, fan intensity, etc.
5. Alerts in the form of “notifications” are provided to optimize appliance usage and energy saving which is not available in other similar apps.

### Performance comparison of the proposed system

The proposed system is discussed regarding the previously proposed home automation systems. Several important parameters are considered for performance comparison. For example, the type of devices/sensors used is one of the important factors that indicate the cost-effectiveness of a system as well as the ease of system installation. Similarly, real-time sensors data, sensors data logging for optimization, automatic execution of the user’s set preferences, system recovery, and remote access are among the favorable controls that a user needs for the automated home. Table 2 shows the performance metrics used for the comparison and points out the advantages that the proposed system has over other similar home automation systems.

**Table 2:** Performance comparison of the proposed system with already proposed systems.

Features	Automation systems								
	[6]	[15]	[16]	[17]	[22]	[43]	[44]	[45]	Proposed
App to make home prototype	✘	✘	✘	✘	✘	✘	✘	✘	✔
Device status data logging	✘	✘	✘	✘	✔	✘	✘	✘	✔
Real time sensors data display	✔	✘	✔	✔	✔	✔	✘	✘	✔
Use of micro-processor (Raspberry Pi)	✔	✘	✔	✘	✘	✔	✘	✘	✔
Internal network in case of gateway failure	✘	✘	✘	✘	✘	✘	✘	✘	✔
Sensors recent state recovery	✔	✘	✔	✘	✘	✘	✘	✘	✔
Light and fan intensity control using pulse wave modulation	✘	✘	✘	✘	✘	✘	✘	✘	✔
Use of ordinary electrical appliances	✔	✘	✔	✘	✘	✘	✘	✘	✔

All the features and functionality described in Table 2 makes the proposed system unique from the rest of the systems. Designing the prototype of one’s own home and setting each device according to the room design makes it easier for a user to operate the electronic device easily.

## Conclusion

This study puts forward the design and full implementation of a home automation system that is low-cost, energy-efficient, and user-friendly. Comprised of admin and user modes, the proposed system can give the user the complete control to design the smart home to the user’s actual home. An intuitive and interactive GUI based smartphone app is designed in Android Studio to control the appliances in the smart home. With the drag and drop option introduced in the smartphone app, home can be designed within a few minutes. The user can control the appliances using local and remote modes whereby local mode does not need the internet and can operate via Raspberry Pi server alone. Microsoft Azure cloud database server is implemented that provides the user access to their home appliances when they are away. The proposed system performs data logging, as well as, the display of the live status of all the installed appliances/sensors. For the current project, the general-purpose electrical appliances have been utilized instead of IP-based sensors that are expensive to purchase and time-consuming to install. Data logging ensures the sensors recovery to their recent state, in case of electricity break down and system gateway failure.

The proposed systems enable the optimized usage of the electrical appliances by displaying the current status of the appliances and by sending “notifications” to the user if an appliance is running over two hours. Besides, the user can set his preferences of temperature and the system turns on and off the ventilation fans and AC to meet those requirements. The proposed system is user acceptance tested and operational in many homes at the moment. However, due to confidentially and security concerns of the users the actual home implementation is not shown in the paper. Instead, a small test bench is used to show the overall implementation and working methodology of the proposed home automation system. It provides simplicity, flexibility, reliability, and a low-cost system that is affordable to middle-class families too. We intend to improve the system by adding more features like heating water management, voice control commands, and employing solar panels to provide energy for the smart home in the future.
